# Evaluation of Catmint Oil and Hydrogenated Catmint Oil as Repellents for the Flour Beetles, *Tribolium castaneum* and *Tribolium confusum*

**DOI:** 10.1673/031.011.12801

**Published:** 2011-09-27

**Authors:** Frank H. Arthur, Emily A. Fontenot, James F. Campbell

**Affiliations:** USDA-ARS-Center for Grain and Animal Health Research, 1515 College Avenue, Manhattan, Kansas, USA

**Keywords:** insecticides, treated surfaces, behavior

## Abstract

Catmint oil and hydrogenated catmint oil were evaluated as repellents for adult *Tribolium casteneum* (Herbst) (Coleoptera: Tenebrionidae), the red flour beetle, and *T. confusum* (DuVal), the confused flour beetle, using both a traditional method of visual assessment of distribution and a video recording method to determine movement patterns of individual insects. Visual assessments of distribution using groups of adults showed that the hydrogenated catmint oil was more effective than the pure catmint oil, but there was no significant difference between species. However, when repellency was measured using single insects and the visual recording system, both oils were significantly more repellent to *T. castaneum* than *T. confusum* at the concentrations evaluated in the study. Avoidance movement and change in direction when *T. castaneum* encountered the repellent were observed. Results indicate that repellents may be more accurately assessed using single insects rather than groups of individuals, and simple visual observations of distribution may be less sensitive in measuring repellent efficacy. Procedures for utilizing a video system are described as models for future evaluations of repellents for stored-product beetles.

## Introduction

In recent years there have been many published studies in which natural plant extracts, also referred to as botanicals, essential oils, and natural products, have been evaluated for activity against stored-product insects (Rozman et al. 2007; Rajendran and Sriranjini 2008). The effectiveness of these compounds often varies depending on the method of testing, the specific compound, and the target pest species. While some plant extracts have shown contact toxicity or fumigant activity, there has been little commercialization of these products in developed countries, for a variety of reasons, including difficulty in producing large quantities of plants necessary to obtain enough of the extract for widespread use. Costs associated with the regulatory registration processes for use of plant products as insecticides may also limit commercialization.

Natural plant products are used commercially as repellents for biting insects. Some plant compounds also exhibit repellent activity against stored-product insects, in addition to direct toxicity and sub-lethal effects. Some examples include garlic (Rahman and Montoyama 2000), azadiractin, an extract of the seed from the neem tree (Muda and Cribb 1999; Hou et al. 2004; Wong et al. 2005), diethyl-*m*-toluamide DEET (Hou et al. 2004), pea products (Fields et al. 2001; Mohan and Fields 2002; Kumar et al. 2004), and citronella oil (Wong et al. 2005). In many of the tests cited above, the experiments involved repellent activity on whole grains rather than on treated surfaces. There have been few tests conducted in recent years in which repellents have been applied to a surface and evaluated against stored-product insects, although this may represent a more desirable target for these compounds since it does not involve treating the food material.

Essential oil repellents may have potential use in the stored-product market, particularly as surface treatments to the floors of food storage facilities as a barrier treatment. Members of the catmint genus *Nepeta* in the family Laminaceae produce essential oils that are effective repellents of mosquitoes. A commercially-available oil from the catmint plant *Nepata cataria* L. (Lamiales: Lamiaceae) and the hydrogenated catnip oil applied at 15% active ingredient by weight gave 4 to 7 hours of protection from the blackfly *Simulium decorum* and mosquitoes from the genus *Aedes* (Spero et al. 2008). This new product has not been evaluated for activity against stored-product insects.

Evaluations of repellents have been done by treating one side of the arena with a repellent, placing a group of adults of one of these species inside the arena, and at selected post-introduction periods recording the number of individuals on the treated and untreated portions of the arena (Halliday et al. 1986; Halliday and McGovern 1987). Two species that have been evaluated this way are *Tribolium castaneum* (Herbst) (Coleoptera: Tenebrionidae), the red flour beetle, and *Tribolium confusum* (DuVaL), the confused flour beetle. Adult *T. confusum* do not fly, and adult *T. castaneum* do not normally fly unless temperatures are above 25 to 30° C so they are more likely to contact treated surfaces. Therefore, the objectives of our study were to: 1) evaluate two different repellents for efficacy, using a traditional method of efficacy assessment, and 2) develop a method to rapidly assess repellent efficacy within a short time period, using a video recording system.

## Materials and Methods

### Traditional method of visual assessment

Repellents used were catmint oil E11187-32-1 YG (CO), and hydrogenated catmint oil E110304-52-2 YG (HCO). The hydrogenated oil is enriched in the repellent, dihyronepatalacetone. Both oils have been shown to be effective against biting insects (Spero et al. 2008). They were supplied by E.I. DuPont de Nemours and Company (www.dupont.com). These compounds were stored at ambient laboratory temperature (ca. 27° C) for several weeks. Concrete treatment arenas were constructed using the bottom portion of a standard 90 mm plastic Petri dish, which has a measured surface area of about 62 cm^2^ and the dry concrete patching material, Rockite (www.rockite.com). This type of arena has been used in a number of previous studies to create a smooth concrete surface (Arthur 2007). The dry patching material was mixed with water in an approximate ratio of 3,200 g Rockite in 1,600 ml of water to create a liquid slurry, and each of 20 dishes was filled to a depth of approximately 1.25 cm to create individual treatment arenas. The arenas were allowed to harden overnight before being used. After the arenas hardened a line was drawn down the center using a marker pen to separate the arena into to approximately equal halves.

Both repellents were diluted by mixing 1 ml of each one in 2, 5, 10, or 25 ml of 99% isopropyl alcohol (by volume), to create a series of dilutions at 50, 20, 10, and 4% strength by volume, one set of dilutions for each repellent CO and HCO. For each concentration and repellent combination, arenas were treated by spraying 1 ml of the diluted solution onto the left half of each arena, using a Badger 100 artists' airbrush (Badger Air-Brush Company, www.badgerairbrush.com). The other half of the dish was left untreated. Two dishes each were treated with each of the four concentrations of the two repellents and four additional untreated arenas were included as controls, which gave a total of 20 dishes (10 for each *Tribolium* spp.). All of the treated arenas were allowed to dry for approximately 1 hour and then either ten 1–2-week-old adult *T. castaneum* beetles or ten 1–2-week-old adult *T. confusum* were placed in the arenas. All arenas were held on a table in a laboratory, with a photoperiod of 9:15 L:D, at approximately 25° C and 40% RH. After 24 hours, the number of beetles in each half of the dishes was recorded daily for 6 days post-treatment. This entire procedure was repeated three times, for a total of three replicates for each treatment combination and four replicates for controls.

Data were analyzed using the General Linear Models of the Statistical Analysis System (SAS 2007) to determine significance of the main effects repellent, concentration, insect species, and day post-treatment. Data were transformed by square root to normalize variances for the statistical analysis, but actual mean values are reported in Table 1. Because all of the post-treatment observations were made on the same set of treatment arenas, the day post-treatment was considered as a repeated measure, and accounted for in the statistical analysis.

### Video recording method

The same catmint oil formulations and arenas as described above were used in this experiment. In these experiments, a ‘spot’ of compound (200 µl of repellent or control oil dispensed using a micro-pipetter) was applied to the concrete along the outer circumference of the arenas. This amount created a circular treated area of approximately 4.9 cm^2^, or 7.0% of the total area of the concrete arena. Separate experiments were conducted using 0.5%, 1.0%, and 2.0% concentrations of repellents CO and HCO, with 200 µl isopropyl alcohol used as a control treatment. A single 1–2-week-old adult *T. castaneum* was put in the experimental arena, and then the arena was placed under a video camera (Ethovision, Noldus Information Technology Inc, www.noldus.com) and beetle activity was recorded for five minutes. A second experiment was conducted using the same concentrations and arenas as described above, but visual observations were made during 5-minute periods at each of 5 days post-treatment, starting 24 hours after the beetles were placed on the arena. The arenas were held at the environmental conditions described above. The presence or absence of beetles in the treated spot was recorded. For each treatment and control the process was replicated 10 times with *T. castaneum*, using new individual adults in new treated arenas each time, and then 10 replicates were done with *T. confusum*, again using new individuals and treated arenas each time. This entire procedure was repeated twice as two blocks with a total of 20 replicates.

**Table 1.**
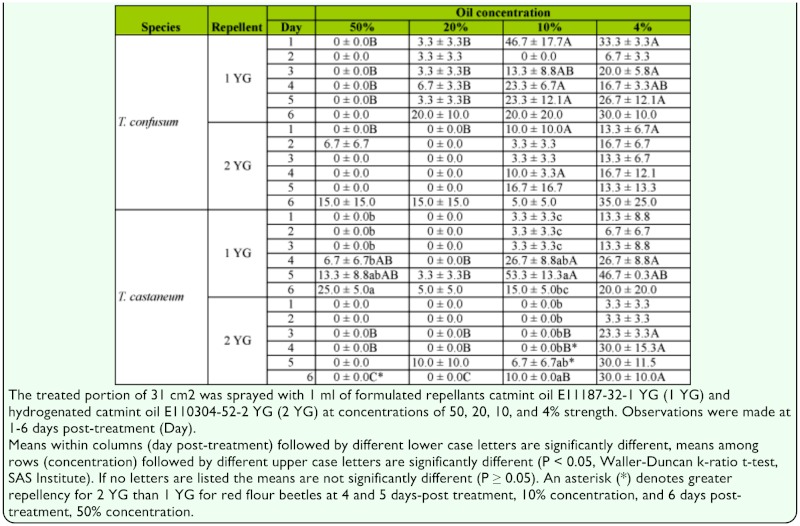
Percentage (mean ± SEM) of adult *Tenebrio confusum* and *T. castanaum* on the treated half of a 62 cm^2^ concrete treatment arena.

The video of each individual adult of each species was viewed and the number of visits each individual beetle made into the treated area and the occurrence of reversals in movement when encountering edge of treated area was counted and recorded. A visit was defined as completely passing through the treated spot without any reversal of direction or avoidance behavior. The same procedure was followed for the controls treated with only isopropyl alcohol. Observations from visual observation and the video recordings were analyzed by the t-test of SAS, with the number of visits by individual beetles into the spots on the arenas treated with repellent as the variable of analysis.

## Results

### Traditional method of visual assessment

The presence or absence of beetles on the left-hand side of the dish in the control arenas appeared to be random. An analysis was done for the untreated controls, and there was no significant difference regarding the presence of the beetles on the left-hand side of the arena versus the right side of the arena with respect to treatment (left side treated with isopropyl alcohol versus the right side that was untreated) or with time (day post-treatment) (F = 4.6, df = 1, 5, P = 0.08; F = 1.8, df = 5, 45, P = 0.14). There was a significant difference between species (F = 47.2, d. f = 1,5, P < 0.01). Data were combined, and the percentage of *T. castaneum* on the left side of the arenas versus the percentage of *T. confusum* on the left side was 55.4 ± 1.9 versus 41.2 ± 3.0, which was significantly different at P < 0.01. However, as will be shown below, this same difference did not occur between species in the arenas treated with the repellents, hence the data for untreated controls were eliminated from the statistical analysis.

The overall statistical analysis of the percentage of adults on the treated side of the arenas showed a significant effect for concentration (F = 14.5, df = 3, 26, P < 0.01), repellent (F = 6.1, df= 1, 26, P = 0.02) and the repeated measure time (day post-treatment, F = 10.0, df = 5, 210, P < 0.01), but no difference between the two insect species (F = 0.2, df = 1, 26, P = 0.65). Generally, repellency appeared to decline with both decreasing concentration and increasing time after treatment (Table 1), but there was considerable variation in the data. The overall ANOVA test showed a significant effect of time, but there were no significant differences with respect to time for *T. confusum* at any of the concentrations of the catmint oil repellent CO. There were three instances of significant difference among time periods for the hydrogenated catmint oil repellent HCO, as denoted by different lower-case letters in Table 1, however, in one of these there was a greater repellent effect at day 5 post-treatment than at day 6. For *T. castaneum*, there were three instances where there was a significant difference with respect to time for either repellent, as denoted by different lower-case letters (Table 1). Similarly, the ANOVA test showed a significant effect of concentration, but there were only 6 out of a possible 30 instances for both species where there were differences among the concentrations for observations made at a particular day post-treatment (Table 1, denoted by different upper-case letters). Finally, the ANOVA indicated a difference between the two repellents, but when the means were compared for each species at each concentration and exposure period, there were no significant differences between repellents for *T. confusum.* The only significant differences between repellants for *T. castaneum* occurred at post-exposure periods 4 and 5 at the 10% concentration and post-exposure period 6 at the 50% concentration (Table 1, asterisks), and indicated a greater repellent effect for HCO compared to CO.

These results show the limitations of testing repellents using the standard method of treating one half of an experimental arena with the repellent, and then making observations of insect distribution at selected time periods. The inherent variation in making a single observation at one time period with a relatively small number of insects limits the accuracy of these data.

### Video recording method

The initial results of the video recording method using HCO repellent at a concentration of 1% were analyzed by t-test, using individual beetles as replicates and recording the number of times each individual was in the treated spot during the 5-minute recording interval (10 replicate individuals of each species). The average number of visits into the treated spot by *T. castaneum* and *T. confusum* was 0.5 ± 0.2 and 3.0 ± 0.6, respectively, indicating that *T. castaneum* exhibited a stronger avoidance response to the treated spot than *T. confusum.* In the untreated controls individual *T. castaneum* made an average of 2.5 ± 0.5 visits into the spots treated with isopropyl alcohol only compared to 3.7 ± 1.3 visits for *T. confusum*, and there was no significant difference between species (P = 0.40). In the tests with CO repellent, individual *T. castaneum* averaged 0.2 ± 0.2 visits into the treated spots compared to 1.7 ± 0.4 visits by the individual *T. confusum*, again indicating greater avoidance response by *T. castaneum* (P < 0.01). Visits made by both species into the repellent spots were significantly lower than the corresponding alcohol spot in the untreated controls (P < 0.01).

In the tests whereby individual beetles were observed daily for 5 days, the number of times that *T. castaneum* and *T. confusum* visited the spot was 1.2 ± 1.5 and 1.6 ± 1.5, with no significant difference between (P = 0.60). This manner of evaluation was similar to what was done using the traditional method, and produced the same result of no difference between the two beetle species. For the series of tests with 1% CO repellent that utilized the video recording, *T. castaneum* averaged 0.3 ± .02 visits into the treated spot, which was significantly less (P < 0.01) than the averages visits of 1.7 ± 0.5 for *T. confusum.* When the average visits during the 5-minute videos were compared for *T. castaneum* and *T. confusum* exposed to each of the repellents, there was no significant difference between the two repellents for either species (P = 0.20 and P = 0.06 respectively).

For the trials with the 0.5% concentrations and *T. castaneum*, average visits of individuals into the treated spots were 0.8 ± 0.4 for CO repellent and 0.5 ± 0.2 for HCO repellent, with no significant difference between the two (P = 0.56). In the trials with the 2% concentration and *T. confusum*, individual visits into the treated spot averaged 1.0 ± 0.4 for CO and 2.4 ± 0.4 for HCO, indicating greater avoidance response to CO repellent for this species and concentration (P < 0.03).

The viewing of the video files with the tests conducted with the 1% concentrations showed that *T. castaneum* exhibited reverse directional movement 2.6 ± 0.5 times when it encountered the spot treated with HCO repellent, in contrast to 0.9 ± 0.2 instances of reverse directional movement for *T. confusum.* There was a significant difference (P < 0.01) between the two species. For the CO repellent, *T. castaneum* reversed direction 2.3 ± 0.4 times, in comparison to 1.0 ± 0.3 times for *T. confusum*, which again showed a significant difference (P < 0.03) between the two species. The greater sensitivity of *T. castaneum* relative to *T. confusum* was evident by their avoidance of the treated area and the reverse directional movement when they approached the treated area. There was no difference in reverse directional movement of either species with respect to the two repellents (P > 0.05).

## Discussion

The results from the traditional method indicated that both repellents CO and HCO were repellent to both species, but there was no difference regarding the level of avoidance behavior between the two species. However, part of the reason for the lack of a significant difference between species could relate to a lack of resolution that can occur when conducting tests with groups of insects. In previously published tests in which repellents were evaluated by treating one side of an arena with a repellent, placing a group of 10 adult *T. confusum* inside the arena, and making counts of how many individuals were on each side of the arena at selected intervals post-treatment, the authors did not analyze their data by analysis-of-variance and mean separation tests (McGovern et al. 1984; Halliday et al. 1986; Halliday and McGovern 1987). Instead, they developed a classification scheme based on mean percent repellency, and there were no estimates of variation about the mean. In addition, in these studies cited above, tests were conducted on the same experimental arenas at different time intervals post-treatment, but apparently were not considered as a repeated measure. Analyzing data as a repeated measure lowers the denominator degrees-of-freedom, thereby making it harder to obtain significance. Mohan and Fields (2002) developed a rapid-test method to assess repellent efficacy in bulk grain, using groups of insects for their studies.

The use of the video recording system required treatment of a small portion of the experimental arenas. However, the rapid-test method utilized in the video experiments, whereby observations were conducted on individual beetles for a continuous 5-minute time period, showed *T. castaneum* was more susceptible than *T. confusum* to either the catmint oil or the hydrogenated catmint oil repellents. This was in sharp contrast to the results for the “standard” method of testing repellent efficacy.

Observations of the traditional tests with 1% concentration of both the catmint oil and the hydrogenated catmint oil repellents indicated that adult *T. castaneum* would generally travel around the circumference of the experimental arena until they encountered the spot treated with the repellents, and then turn and reverse direction. This behavior would be repeated when *T. castaneum* traveled around the circumference of the arena and encountered the treated spot from the other side. In contrast, when adult *T. confusum* encountered the area with the 1% repellent solutions, some of the individuals would not exhibit this reverse directional movement or avoidance behavior. Video recordings allowed for repeated viewings and quantitative confirmation of this behavioral difference between the two species, which has not been previously recorded.

Watson and Barson (1996) conducted tests in which individual *Oryzaephilus surinamensis*, the sawtoothed grain beetle, were exposed on experimental arenas in which one side was treated with repellents or insecticide. The reason cited for using individual *O. surinamensis* rather than groups was to avoid any possible tendencies for groups to clump or aggregate, thus biasing the results. Papachristos and Stamapoulos (2002) conducted studies in which individual inseminated female *Acanthosclides obtectus*, the bean weevil, were exposed singly in experimental arenas to evaluate repellent activity and ovicidal effects of been seeds treated with various essential oils. In our experiments, we used individual adults and were therefore able to show a difference between *T. castaneum* and *T. confusum* in their response to the repellents. In addition to variation in response when using groups, perhaps some behavioral responses in groups of *T. castaneum* and *T. confusum* versus individuals accounted for the lack of a difference in the response of the two species to the catmint oil and hydrogenated catmint oil repellents.

In conclusion, this rapid-assessment method utilizing a video recorder could be expanded for future studies involving repellent evaluations. One advantage to video taping is that you can fast forward through the tape and stop at beetle encounters with the repellent. It could be done in less time then direct visual observation, and because of the ability to go back and forth and speed up and slow down the tape, movement and avoidance behavior can be recorded more accurately. Although a human observer could collect data by observing the beetles for a given time period, the video recording provides a record which can be saved on a computer and repeatedly viewed for additional observation and analysis. These video files also enable the automation of data collection using a tracking system such as the Noldus Ethovision program. A final benefit is that the use of spots of repellent compounds enables assays to be conducted using much less experimental material than treating half of a dish.

## Abbreviations:

**CO**,: catnip oil;
**HCO**,: hydrogenated catnip oil
